# Gas Sensing Properties of Cobalt Titanate with Multiscale Pore Structure: Experiment and Simulation

**DOI:** 10.3390/s20061787

**Published:** 2020-03-24

**Authors:** Mingchun Li, Baoting Wang, Aili Tao, Shengfei Li

**Affiliations:** College of Materials Science and Engineering, Shenyang University of Technology, Shenyang 110870, China; wbtjms@163.com (B.W.); taoaili1994@163.com (A.T.); 13324075512@163.com (S.L.)

**Keywords:** gas sensors, diffusion-reaction model, multiscale pore structure, cobalt titanate, control mechanism

## Abstract

A diffusion-reaction coupled model was presented to investigate the effects of multiscale pore structure characteristics on gas sensing properties. A series of CoTiO_3_ powders with different pore size distributions were fabricated by sol-gel method. Experimental results on cobalt titanate thick films show that a well-defined multiscale pore structure is particularly desired for the improvement of sensing performance, instead of just increasing the specific surface area. The theoretical responses of sensing elements with different pore size distributions were derived and compared with experimental data on CoTiO_3_ sensors exposed to ethanol. The calculated sensitivities considering the influence of pore size changes were also found to be in agreement with the experimental results. A dimensionless Thiele modulus *Th* was introduced for assessing the critical point corresponding to the transformation from surface reaction-controlled sensitivity into diffusion-controlled sensitivity.

## 1. Introduction

Gas sensors based on semiconducting metal oxides have been regarded as the most versatile method for gas detection, due to high sensitivity, good stability, simple fabrication process, and wide variety of applications [1,2,3]. In the presence of target gas, changes in the electrical conductance of the metal oxide materials are caused by the physical adsorption of target gas followed by a chemical reaction between target gas and surface chemisorbed oxygen. Thus, gas diffusion through the porous sensing layer plays an important role for gas sensing properties. Considerable effort has been directed towards optimizing the structural and textural properties of semiconducting metal oxides [4,5,6,7,8,9,10,11]. Among the developed innovative structures, hierarchical structures with morphology and high porosity endow the sensing materials with greater response owing to their high surface area and fast gas transport [12,13,14]. For example, Guo et al. elucidated that hierarchical porous hollow TiO_2_ nanofibers exhibited a remarkable higher sensing performance due to the peculiar properties arising from a large surface/volume ratio [15]. Li et al. reported the synthesis of a ZnO hierarchical microstructure that assembled by single-crystalline porous nanoplates and its superior sensing performance to triethylamine as compared with commercial ZnO nanoparticles [16]. Via a graphene oxide-assisted hydrothermal route, Zhao et al. reported the design and synthesis of tin oxide hierarchical porous nanosheets, which exhibited greatly enhanced ethanol sensing performance as compared with bare SnO_2_ nanosheets [17].

Considering the practical necessity of porous nanostructure for high performance gas sensing, there is extensive literature on the fabrication of porous semiconducting metal oxides with different morphologies, such as porous nanocubes [18], nanoshuttles [19], nanocages [20], nanosheets [21], nanofibers [22], and their enhanced gas-sensing properties owing to the well-defined and uniform porous structures. It is believed that nanostructured cobalt titanate with abundant pores exhibits an improved gas sensing performance as compared with the nanocrystalline CoTiO_3_. Several investigators have reported on the specific synthesis approaches of porous cobalt titanate such as electrospinning method [23], ethylene glycol-mediated method [24], template method [25], and sol-gel method [26]. However, less attention has been paid to the effect of pore size distribution on the gas sensing performance.

Most investigators have mainly focused and made great progress on the fabrication methods of novel hierarchical nanostructures [27,28,29,30], and their observations seem to agree with the general trend that higher sensitivity is to be expected for larger surface/volume ratio and uniform pore structure [1,5,31,32]. However, pore size and specific surface area are correlated in sensor materials with potentially opposite effect. Decreasing the pore size under similar porosity leads to both higher surface/volume ratio and larger diffusion resistance, which can have a negative effect on sensing performance. In addition, target gas diffuses within the pores and reacts simultaneously on the surface of the sensing materials, leading to the dynamic changes in both the local gas concentration and, consequently, the electrical resistivity within the porous layer, confirming a strong dependence of gas sensing performance on the diffusion reaction coupled effects related to pore size distribution. Hence, the authors argue that a well-defined multiscale pore size distribution is more favorable for higher sensitivity than just increasing the surface/volume ratio.

In general, researches have related the diffusion-reaction model [33] with conduction or sensitivity of the semiconductor gas sensor most focused on the gas diffusion within a simplified structure with uniform pore size. For example, by providing that the semiconductor layer was macroscopically homogeneous, Gardner proposed a nonlinear diffusion-reaction model of a semiconductor gas sensor to cover a nonlinear reaction process coupled with linear diffusion [34]. Assuming that Fick’s law is applicable to porous gas sensing materials with surface reaction, Lu et al. formulated a diffusion-reaction model for conductance response in metal oxide gas sensing thin films based on the measured porosity and a calculated uniform pore size [35]. By using a simplified porous structure consisting of uniform pores with a representative radius r, Sakai et al. solved a gas transport equation under steady-state conditions to obtain the concentration profiles of target gas inside a sensing film to estimate the gas sensitivity [36]. Under steady-state condition, Miao et al. provided a quantitative explanation for the sensitivity improvement of monolayer film made from CuO nanosheets with a model considering the coupled Knudsen diffusion and first-order surface reaction [37]. Based on diffusion equations containing a linear term related to the reaction processes and a constant diffusion, a mathematical model for gas sensing thin film semiconductor at an internal diffusion limitation for non-steady-state conditions was presented and solved analytically by Selvaraj et al. [38]. Wang et al. concluded that controlling microstructure and exploring the interaction effects between gas diffusion and surface reaction to the sensing mechanism would be necessary for improving the sensitivity and response to different gases [39]. Thus, it is still of great importance to establish a general coupled model that reflects the interaction among pore size distribution, mass transfer, surface reaction and the sensing properties.

The objectives of this research are to investigate the effects of multiscale pore structure characteristics and the coupled diffusion-reaction on the gas sensing properties. CoTiO_3_ powders with different pore structure characteristics were fabricated following the sol-gel method, and the corresponding sensing performances were experimentally investigated. A mathematical model that considers the multiscale coupling of the pore structure characteristics with the diffusion behavior and surface chemical reaction was established and fitted to the gas sensing measurements data. The quantitative limit for judging the transformation of the sensing mechanisms was discussed.

## 2. Materials and Methods

Cobalt titanate with multiscale pore structure was synthesized following the sol-gel method using tetrabutyl titanate as the titanium source and cobaltous nitrate hexahydrate as the cobalt source. The diethanolamine (C_4_H_11_NO_2_, DEA) was used as a structure-directing agent of porous structure. In a typical synthesis, first, 3.88 g of cobaltous nitrate hexahydrate was dissolved in 6 mL of deionized water forming clear solution. Then, 13.3 mmol tetrabutyl titanate and 2 mL diethanolamine were mixed in 120 ml of absolute ethanol to obtain a complex solution. After 20 min, the prepared cobalt nitrate solution was added dropwise to the complex solution under stirring and the pH value of the mixed solution was adjusted to 3.5 using acetic acid. The mixed solution was stirred at 300 r/min until the gel was formed. Finally, the obtained gel was dried at 70 °C for 8 h and calcined at 600 °C for 2 h. Synthesized samples were, respectively, denoted as CT0, CT1, CT2, and CT3 according to different molar ratios of DEA/Ti/Co of 0:1:1, 0.9:1:1, 1.75:1:1, and 2.8:1:1.

The crystal phases of the as-obtained products were characterized with X–ray diffractometer (Shimadzu, 7000S, Tokyo, Japan) using Ni–filtered Cu Kα radiation. The morphology of samples was characterized using a field emission scanning electron microscopy (Hitachi, SU8010, Tokyo, Japan). The pore structure characteristics including specific surface area and pore size distribution were obtained by nitrogen adsorption−desorption method (Gold APP, Vsorb 2800P). The specific surface area was determined by a multipoint Brunauer–Emmett–Teller (BET) method using the adsorption data, and the pore size distribution was analyzed via the Barret–Joyner–Halender (BJH) method.

**Figure 1:**
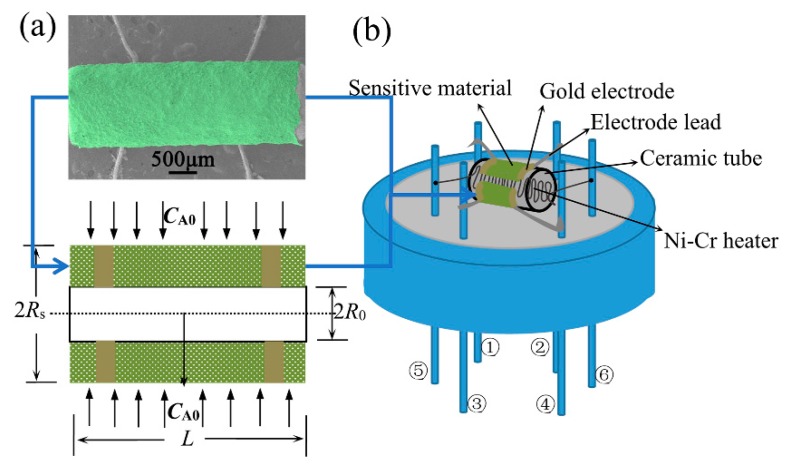
Physical model and schematic diagram of gas sensor. ①, ②, ③, ④ measuring electrodes; ⑤ and ⑥ heating electrodes. (**a**) SEM image and physical model of gas sensor; (**b**) Schematic diagram of sensor structure.

For the as-prepared samples, thick film gas sensors were prepared on an alumina ceramic tube attached with a pair of Au electrodes and Pt wires. A Ni-Cr alloy heating wire was inserted into the tube to form a heater. The schematic diagram of the CoTiO_3_ sensor and the corresponding physical model are shown in Figure 1. Then, the sensing behaviors of the synthesized samples were analyzed with the fabricated sensors using a static measurement system (Weisheng Electronics, WS-60A). The sensor device and the measurement system equipped with a 20 L chamber were reported by our previous work [26]. Within a typical gas sensing test cycle, first, clean dry air was introduced into the sensing chamber to record the baseline of the whole test. After a stable voltage baseline at working temperature was achieved, 0.0053 mL ethanol (corresponding to 100 ppm ethanol concentration) was injected into an evaporator, which was instantly evaporated and uniformly distributed throughout the test chamber by program heating and stirring. The exposure time for the test gas was 5 minutes. Finally, the sensor began to recover in clean air again for 5 min.

## 3. Results

**Figure 2:**
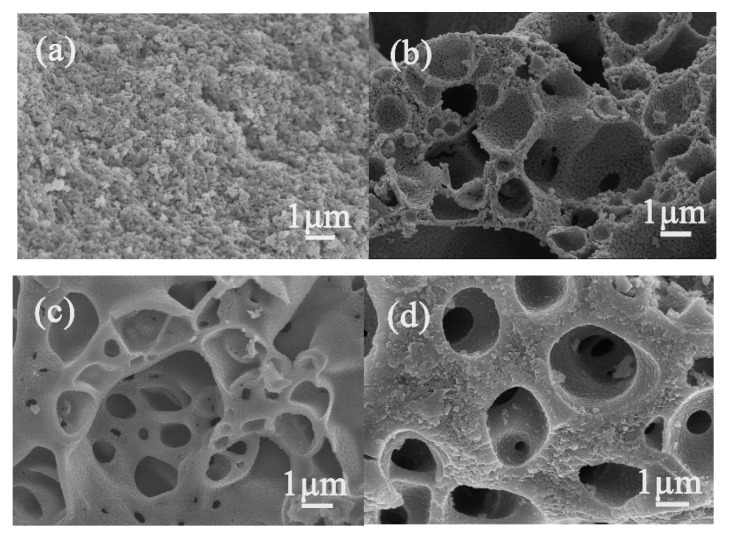
SEM images of the as-prepared products. (**a**) Sample CT0; (**b**) Sample CT1; (**c**) Sample CT2; (**d**) Sample CT3.

**Figure 3:**
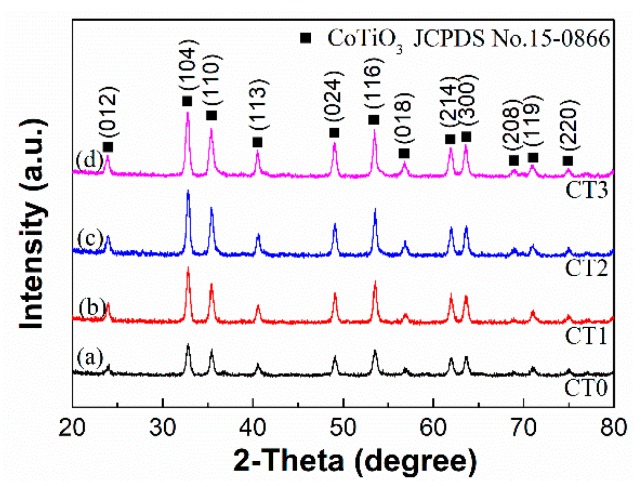
XRD patterns of samples CT0, CT1, CT2, and CT3.

The representative SEM images and XRD patterns of the as-prepared products obtained at different molar ratios of DEA/Ti/Co are displayed in Figure 2 and Figure 3. Sample CT0, prepared without DEA, illustrates a compact structure of grain accumulation, as shown in Figure 2a. Large macropores with a size of approximately 0.5 to 7 μm distributed in the porous matrix were observed in sample CT1 obtained at a molar ratio of DEA to Ti/Co of 0.9:1:1 in Figure 2b. These large macropores are interconnected through the small pores between grains on the walls, resulting in a multiscale pore structure that has a relatively large adsorption capacity and a higher utilization efficiency of its internal surface area. Figure 2c also shows a similar macroporous framework over a pore size range of 0.2 to 6 μm, but the smooth walls of these large pores are formed by close-packed nanocrystals at the molar ratio of DEA to Ti/Co of 1.75:1:1 (CT2). As the molar ratio of DEA to Ti and Co reaches 2.8:1:1 (CT3), only a few discrete large pores separated from each other by thick pore walls can be seen within the as-prepared products, as shown in Figure 2d.

The diffraction peaks at 2θ of 23.92°, 32.81°, 35.39°, 40.52°, 49.02°, 53.49°, 56.86°, 61.91°, 63.54°, 68.78°, 71.38°, and 74.87° corresponding to the rhombohedral CoTiO_3_ all can be observed from curves (a)–(d) in Figure 3, which are in agreement with the standard JCPDS No. 15-0866. The XRD peaks of sample CT0 appear much lower than those of samples CT1, CT2, and CT3, suggesting the small crystal size and low crystallinity of sample CT0.

**Figure 4:**
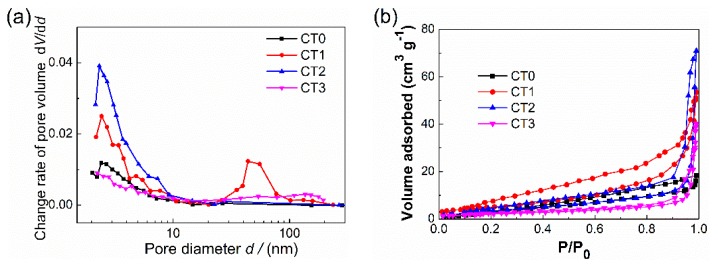
Pore structure characteristics of samples CT0, CT1, CT2, and CT3. (**a**) Pore size distributions; (**b**) Isothermal absorption and desorption curves.

To further confirm the inner pore structure characteristics, the corresponding pore size distributions and nitrogen adsorption and desorption isotherms of samples CT0, CT1, CT2, and CT3 are presented in Figure 4. As Figure 4a shows, the pore size distributions of the as-prepared products all demonstrate a mesoporous characteristic in the pore size range from 2 nm to 10 nm. In particular, the microscopic pore structure of CT2 corresponds to the highest peak of change rate of pore volume and the corresponding specific surface area reaches 46.5 m^2^/g. Within a pore size range from 2 nm to 200 nm, an obvious bimodal distribution can be observed from sample CT1, and the most probable pore sizes are about 2.6 and 50 nm, respectively. Considering the large macropores (0.5 to 7 μm) contained in the porous matrix, as shown in Figure 2b, sample CT1 has a multiscale pore structure characteristic. The specific surface area of sample CT1 is only 29.4 m^2^/g, while the pore volume reaches 0.125 cm^3^/g, which is 1.62 times greater than that of the sample CT2. It can be seen from Figure 4b, all the samples show a similar Type-Ⅲ adsorption isotherms and increase rapidly at high relative pressure *P*/*P*_0_ > 0.8 except for sample CT0, indicating the presence of large pores within samples CT1, CT2, and CT3. 

**Figure 5:**
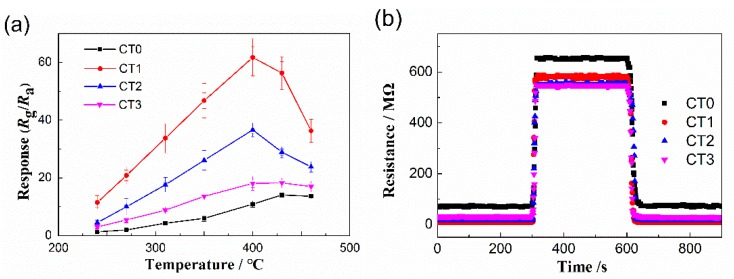
Gas sensing performances of the as-prepared products. (**a**) Gas sensitivity; (**b**) Response and recovery curves at 400 °C.

The sensitivities of porous cobalt titanate sensors based on the as-prepared products to 100 ppm ethanol were measured at working temperature ranging from 240 °C to 460 °C. The sensitivity of the sensor at a certain working temperature was defined as *S* = *R*_g_/*R*_a_, where *R*_g_ and *R*_a_ are the sensor resistances in target gas and in air obtained at this working temperature, respectively. The response time was defined as the time required for the sensor to reach 90% of the final equilibrium value following an injection of the test gas, while the recovery time was defined as the time required for the sensor to reach 90% of the total resistance change as compared with the baseline value in air after releasing the test gas. As shown in Figure 5a, when the working temperature reaches 400 °C, the gas sensor based on the multiscale porous CoTiO_3_ (CT1) shows the highest sensitivity (61.75) to 100 ppm ethanol, which is 5.66 times and 1.68 times as large as that of CT0 and CT2, respectively. This demonstrates that the multiscale pore distribution has great advantages for gas sensing properties due to the effective interconnectivity among macropores (0.5 to 7 μm), large-scale mesopores (50 nm), and small-scale mesopores (2.6 nm), which significantly increases the gas transmission rate and effective utilization of surface area. The response and recovery curves of the sensors based on the as-prepared CoTiO_3_ with different pore structures toward 100 ppm ethanol at 400 °C are shown in Figure 5b. According to the definition of response and recover times, the response times of samples CT0, CT1, CT2, and CT3 are 12, 7, 10, and 9 s and the corresponding recovery times are 29, 21, 25, and 20 s, respectively. It can be seen that the response time of the sensor based on multiscale porous sample (CT1) is faster than that of the others.

## 4. Model

### 4.1. Problem Description

A cylindrical CoTiO_3_ thick film sensor subjects to a constant concentration (*C*_A0_) boundary condition. The outer diameter of the porous sensing layer is 2*R*_s_, and the length of which is *L*. The target gas diffuses radially into the porous sensing layer and was consumed by the reactions with the adsorbed oxygen. The forming of oxygen anions and the redox reactions between target gas A and the adsorbed oxygen are shown in the following equations [40,41].

(1) $$O_{2(\mathrm{gas})}+{2e}^{-}↔2O_{\mathrm{ads}}^{-},\;O_{\left(\mathrm{ads}\right)}^{-}+e^{-}↔O_{\mathrm{ads}}^{2-}$$

A+O^−^_ads_ → (AO)_ads_+e^−^, A+O^2−^_ads_ → (AO)_ads_+2e^−^ (2)

When the sensor is exposed to target gas A, the redox reaction makes more electrons go back to the valence band and neutralizes with the holes, leading to a decrease of the conductance of CoTiO_3_ gas sensor. Assuming that the porous sensing layer is a uniform stack of infinitesimally thin sheets, the electric conductance is given by *σ*(*r*), where *r* denotes the radial position in the layer. The conductance of the whole porous sensing layer is then given by integrating *σ*(*r*) over the whole range from *R*_0_ to *R*_s_ [36]. 

According to the redox Reaction (2), there is an opposite relationship between the electric conductance *σ*(*r*) and the consumed mole of target gas A. When the initial conductance in air is denoted by *σ*_0_, the sheet electric conductance at time *t σ*(*r*, *t*) under exposure to target gas A can be given as follows:

(3) $$\sigma (r,t)=\sigma_{0}(1-\alpha G_{A}(r,t))$$

where *G*_A_(*r*, *t*) denotes the exhaustive mole of target gas A over the radial range from *r* up to *r* + Δ*r* (Δ*r* → 0) at reaction time *t*. The sensitivity coefficient *α* transduces the surface reaction into a relative change of electrical sheet conductance. It would be most susceptible to the change of adsorbed oxygen concentration upon exposure to the target gas as reported by Sakai et al. [36]. The coefficient *α* is assumed to be expressed as follows:

(4) $$\alpha =\alpha_{0}\exp(-\frac{E_{a}-Q_{d}}{RT})$$

where *α*_0_ is a pre-exponential constant; *E*_a_ is the apparent activation energy for the transduction processes, which was estimated to be 117 kJ/mol; *Q*_d_ is adsorption heat of gas molecules, which was estimated in the range of 90–110 kJ/mol; and *R* is the gas constant.

The whole resistances of the porous sensing layer in air, *R*_a_, and in target gas, *R*_g_, can be given as follows:

(5) $$\frac{1}{R_{a}}=\int_{R_{0}}^{R_{s}}\sigma_{0}dr,\;\frac{1}{R_{g}}=\int_{R_{0}}^{R_{s}}\left[\sigma_{0}(1-\alpha G_{A}(r,t))\right]dr$$

Then, the sensitivity of the sensor *S* (the ratio of the sensor resistance in target gas to that of in air) can be achieved.

### 4.2. Diffusion-Reaction Model

Consider a cylindrical thin shell of inner radius *r* and outer radius *r* + Δ*r* (Δ*r* → 0) located within the porous layer, the length of which is *L*. The mass change induced by diffusion in the cylindrical thin shell (*m*_d_) can be expressed by:

(6) $$m_{d}=2\pi rL\varepsilon \Delta rD_{\mathrm{Ae}}[\partial^{2}C_{A}(r,t)/\partial r^{2}+(1/r)\partial C_{A}(r,t)/\partial r]$$

where *ε* denotes the porosity; *D*_Ae_ is the effective diffusion coefficient of target gas A within the porous layer, which can be calculated from the Knudsen diffusion coefficient *D*_AK_ [42] and the effective molecular diffusivity *D*_AM_ [43], 1/*D*_Ae_ = 1/*D*_AK_ + 1/*D*_AM_. According to the obtained experimental data of pore structure parameters in this work, *D*_AK_ and *D*_AM_ are expressed as follows:

(7) $$D_{\mathrm{AK}}=\sum_{i=1}^{M}\frac{\varepsilon d_{i}\varphi (d_{i})}{3\tau }\sqrt{8RT/\left(\pi M_{A}\right)},\;D_{AM}=D_{A}\varepsilon /\tau$$

where *d_i_* is the average pore size of pore diameter interval *i*; $\varphi (d_{i})$ is the pore volume percent; *τ* is the tortuosity (2~3); *M*_A_ and *D*_A_ are the molecular mass and molecular diffusion coefficient of target gas A, respectively.

The mass consumed by the redox reaction in the cylindrical thin shell (*m*_r_) can be given by:

(8) $$m_{r}=2\pi rL\Delta rS_{V}\rho \beta (r,t)kC_{A}^{n}(r,t)$$

where *β*(*r*,*t*) represents the fractional occupancy of surface sites by adsorbed oxygen, and *S*_V_ is the specific surface area; *C*_A_(*r*,*t*) denotes the concentration of target gas at radial coordinate *r* and reaction time *t.* The exponent *n* is the order of reaction. The reaction rate constant *k* that depends on temperature can be calculated as follows [36]:

(9) $$k=k_{0}\exp(-\frac{E_{R}}{RT})$$

where *k*_0_ is the pre-exponential factor that depends on substrate and adsorbate. The activation energy of redox reaction (*E*_R_) was estimated to be 20 kJ/mol.

Then, the mass balance for target gas A in the cylindrical thin shell can be given by:

(10) $$D_{\mathrm{Ae}}\varepsilon [\partial^{2}C_{A}(r,t)/\partial r^{2}+(1/r)\partial C_{A}(r,t)/\partial r]-S_{V}\rho \beta (r,t)kC_{A}^{n}=\varepsilon \partial C_{A}(r,t)/\partial t$$

The corresponding initial and boundary conditions are

(11) $$C_{A}(r,0)=C_{\mathrm{in}},\;\beta (r,0)=\beta_{0}$$

(12) $$C_{A}(R_{s},t)=C_{A0},\;{\partial C_{A}/\partial r|}_{r=R_{0}}=0$$

where *C*_in_ is the initial concentration of target gas in air, *C*_A0_ is the target gas concentration in the polluted ambient air, and *β*_0_ represents the initial fractional occupancy of surface sites by adsorbed oxygen.

Aiming to get the concentration profiles of the target gas *C*_A_(*r*,*t*) and gas sensing properties, the established transient partial differential governing equations were rendered discrete using the implicit finite volume method, by which the properties of conservation and diffusion can be analyzed in detail [44,45]. Then, the whole consumed mole of target gas A due to surface reaction over the radial range from *r* up to *r* + Δ*r* at reaction time *t* can be expressed as follows:

(13) $$G_{A}(r,t)=\int_{0}^{t}\beta (r,t)kC_{A}^{n}(r,t)S_{V}\rho 2\pi rL\Delta rdt$$

(14) $$\beta (r,t)=\beta_{0}(1-\sum_{i=1}^{N-1}\beta (r,t_{i}))\exp(-\frac{E_{\mathrm{abs}}}{RT})$$

where *i* (*I* = 1, 2, …, *N*) denotes the number of time increment. The adsorption energy *E*_abs_ was estimated to be in the range of 10–20 kJ/mol [46]. 

The following dimensionless variables were introduced for normalizing the governing equation:

(15) $$\bar{t}=tD_{\mathrm{Ae}}/R_{s}{}^{2},\;\bar{r}=r/R_{s},\;{\bar{C}}_{A}(\bar{r},\bar{t})=C_{A}(r,t)/C_{A0}$$

Utilizing Equation (15), the governing Equation (10) can be rewritten as:

(16) $$[\partial^{2}{\bar{C}}_{A}(\bar{r},\bar{t})/\partial {\bar{r}}^{2}+(1/\bar{r})\partial {\bar{C}}_{A}(\bar{r},\bar{t})/\partial \bar{r}]-(kR_{s}{}^{2}/D_{\mathrm{Ae}}\varepsilon )S_{V}\rho \beta (\bar{r},\bar{t})C_{A0}^{n-1}{\bar{C}}_{A}^{n}(\bar{r},\bar{t})=\partial {\bar{C}}_{A}(\bar{r},\bar{t})/\partial \bar{t}$$

Let $Th=S_{V}\rho kR_{s}{}^{2}\beta (\bar{r},\bar{t})C_{A0}^{n-1}{\bar{C}}_{A}^{}(\bar{r},\bar{t})/(D_{\mathrm{Ae}}\varepsilon )$, *Th* represents the Thiele modulus that describes the relationship between surface reaction rate and diffusion velocity within the porous sensing layer.

### 4.3. Multiscale Pore Size Distribution Model

On the basis of the obtained Barrett–Joyner–Halender results of multiscale pore structure characteristics for the as-prepared cobalt titanate, a probability density function with multimodal distribution was used to construct the pore volume percent of pore diameter *d* as follows:

(17) $$\varphi (d)=\sum_{j=1}^{K}\frac{P_{j}}{d\sigma_{j}\sqrt{2\pi }}\exp[-\frac{{\left(\ln d-\ln\mu_{j}\right)}^{2}}{2\sigma_{j}{}^{2}}],\;\sum_{j=1}^{K}P_{j}=1$$

where *P_j_* denotes the probability of pore size distribution peak *j (j* = 1, 2, …, *K*), *μ_j_* is the location parameter that corresponds to the most probable pore diameter of the *j*-th pore size distribution peak, and *σ_j_* is the distribution parameter.

**Figure 6:**
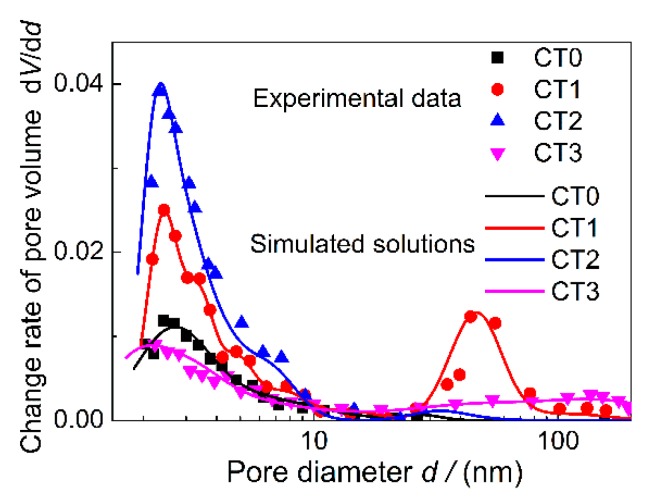
Comparison of the calculated results with the experimental data for pore structure characteristics.

A comparison was made for the simulated solutions of pore size distribution with the Barrett–Joyner–Halender experimental results as shown in Figure 6. The minimum goodness of fit is 0.96.

## 5. Discussion

The pore structure parameters of samples CT0, CT1, CT2, and CT3 and the corresponding effective diffusion coefficients are shown in Table 1. The reaction order *n* in the governing equation was determined using the trial and error method in the range from 0 to 2 based on the experimental data (*n* = 0.87). The other constant parameters in calculation were *L* = 4.0 × 10^−3^ m, *R*_0_ = 4.25 × 10^−4^ m, (*R*_s_ − *R*_0_) = 6 × 10^−8^ m, *C*_A0_ = 4.54 × 10^−1^ mol/m^3^, *α*_0_ = 3.4 × 10^7^ mol^−1^, and *β*_0_ = 0.5, *k*_0_ = 1.1 × 10^−12^ m/s.

**Figure 7:**
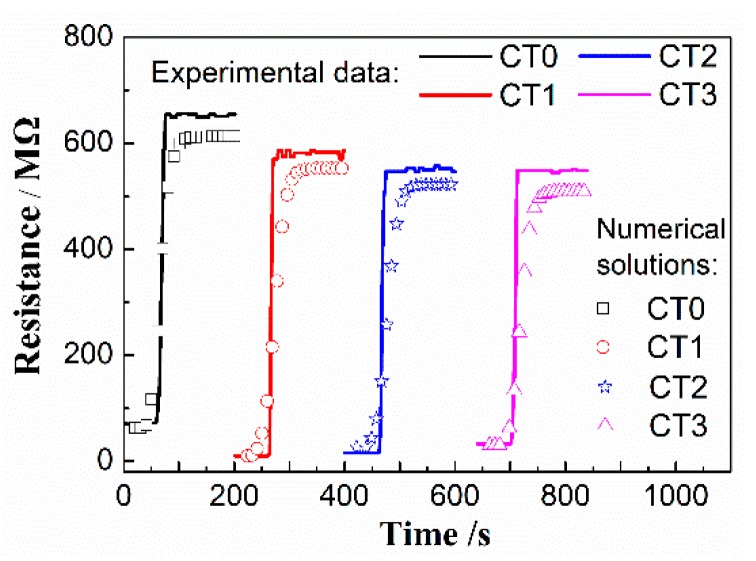
Comparison of the calculated results with the experimental data for resistances of samples CT0, CT1, CT2, and CT3 towards 100 ppm ethanol at 400 °C.

As shown in Figure 7, a comparison was made for the experimental response data of the sensors based on the as-prepared samples with the numerical solutions at the working temperature 400 °C. The symbols in Figure 7 denote the simulated results and the experimental data are represented by solid lines. It can be seen from Figure 7 that the pore structure characteristics have a great influence on the simulated solutions, and sample CT1 showed a maximum calculated sensitivity (*R*_g_/*R*_a_ = 57.68), which is in agreement with the experimental results. However, sample CT0 showed the shortest response time (4 s), which is different from the existing experimental results.

**Figure 8:**
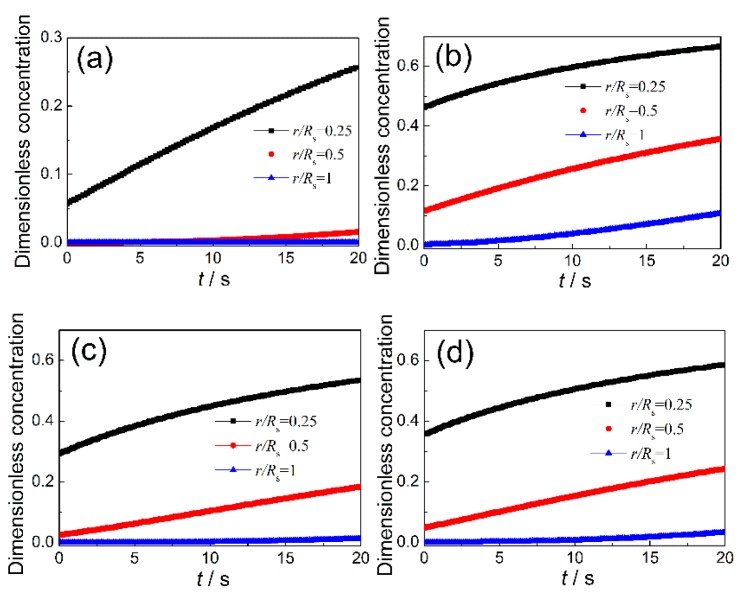
Concentration profiles of target gas at different radial positions of porous sensing layer. (**a**) Sample CT0; (**b**) Sample CT1; (**c**) Sample CT2; (**d**) Sample CT3.

Figure 8 shows the variation of the concentration of target gas at different radial positions of sensing film (*r*/*R*_s_ = 0.25, 0.5, 1) with pore size distribution at the working temperature 400 °C. As seen from Figure 8a, within the response time measured in this work, the diffusion depth of the target gas in sample CT0 was less than half of the sensing layer thickness. As shown in Figure 8b, the concentrations of the target gas within the whole sensing layer of sample CT1, even at the radial position of *r*/*R*_s_ = 1, all increase with test time during the measured response time. This demonstrates that the interconnected multiscale pore structure of sample CT1 has great advantages for gas transmission and most of the surface area within sample CT1 was effectively utilized during the test time, whereas for sample CT2 with the highest specific surface area (see Figure 8c), the concentration of target gas at radial position *r*/*R*_s_ = 0.5 and reaction time 20 s only achieved 18.4% of the initial concentration (*C*_in_). As the sensing properties are determined by the product of the specific surface area and the concentration of target gas, simply increasing the specific surface area can be unfavorable for the sensitivity growth, which is consistent with the experimental results in Figure 5. Although the concentration fields of target gas within sample CT3 are higher than those of sample CT2 (see Figure 8d), the dramatically reduced specific surface area is the main factor for its low sensitivity.

**Figure 9:**
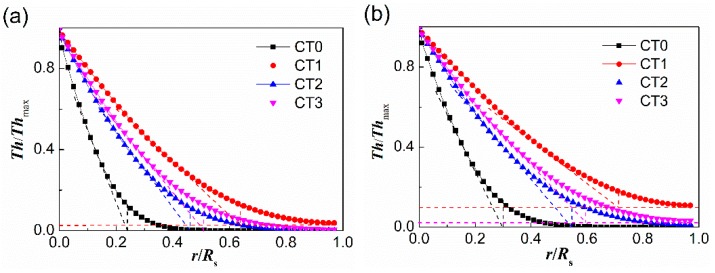
Changes in the ratio of *Th*/*Th*_max_ at different radial coordinate of porous sensors based on samples CT0, CT1, CT2, and CT3. (**a**) Test time 10 s; (**b**) Test time 20 s.

For a simplified comparison of the *Th* curves of samples CT0, CT1, CT2, and CT3 at the working temperature 400 °C in a chart, the same vertical scale *Th*/*Th*_max_ was used. Figure 9a,b portrays the variations of the ratio of *Th*/*Th*_max_ with radial coordinate under different pore structure characteristics at reaction times 10 s and 20 s, respectively. As can be seen from Figure 9, the relative influence of surface reaction and diffusion is diverse at different radial coordinates. The grade change points of the *Th*/*Th*_max_ curves that are determined by the intersection points of the tangent at the point of inflection with the extrapolated horizontal tangent can be used as the critical points to assess whether the sensitivity is controlled by gas diffusion. For samples CT0, CT1, CT2, and CT3, the corresponding critical radial positions (*r*/*R*_s_) at reaction times 10 s and 20 s, obtained from Figure 9, are 0.23, 0.64, 0.47, 0.51 and 0.30, 0.72, 0.54, 0.59, respectively. For sample CT1 with multiscale pore distribution, the gas-diffusion resistance is almost negligible except for the radial position near the boundary wall (*r*/*R*_s_ > 0.8). Combined with the nitrogen adsorption-desorption results shown in Figure 4 and Table 1, it is worth pointing out that the pore size distribution has a significantly effect on both the main limiting step for sensitivity and the effective utilization of the porous layer along the thickness direction. This is useful for sensor design and choosing the most reasonable pore structure.

## 6. Conclusions

Taking into account the multiscale pore structure effects, a diffusion-reaction coupled model describing the gas diffusion, surface chemical reaction, and the corresponding sensitivity of porous gas sensitive sensors was established and verified. The predicted electrical resistivities are in agreement with the experimental results on cobalt titanate thick films with different pore size distributions at a working temperature of 400 °C. The effects of pore structure characteristics on the gas sensing properties and the concentration profiles of target gas were analyzed. The results show that the interconnected multiscale pore structure of sample CT1 has great advantages for the effective utilization of surface area and gas sensing properties. A dimensionless Thiele modulus *Th* was introduced for assessing the relative transformation from surface reaction-controlled sensitivity into diffusion-controlled sensitivity. The developed multiscale coupled model could well lead to improvements in semiconductor materials design and to modifications in the design of thick-film gas sensors.

## Figures and Tables

**Table 1:** Pore structure parameters and the effective diffusion coefficient.

Samples	Adsorption Pore Volume × 10^6^ (m^3^/g)	Specific Surface Area (m^2^/g)	Average Pore Size (nm)	*D*_Ae_ × 10^6^ (m^2^/s)
CT0	0.047	10.2	13.7	0.19
CT1	0.125	29.4	41.43	0.94
CT2	0.077	46.5	5.68	0.51
CT3	0.091	9.75	49.97	0.65
